# Supplementary material for: Active site localization of methane oxidation on Pt nanocrystals
Source: Nat Commun. 2018 Aug 24;9:3422. doi: 10.1038/s41467-018-05464-2 (PMC6109038; doi:10.1038/s41467-018-05464-2)
Supplement: Supplementary file 1 — Supplementary Information [file 41467_2018_5464_MOESM1_ESM.pdf]

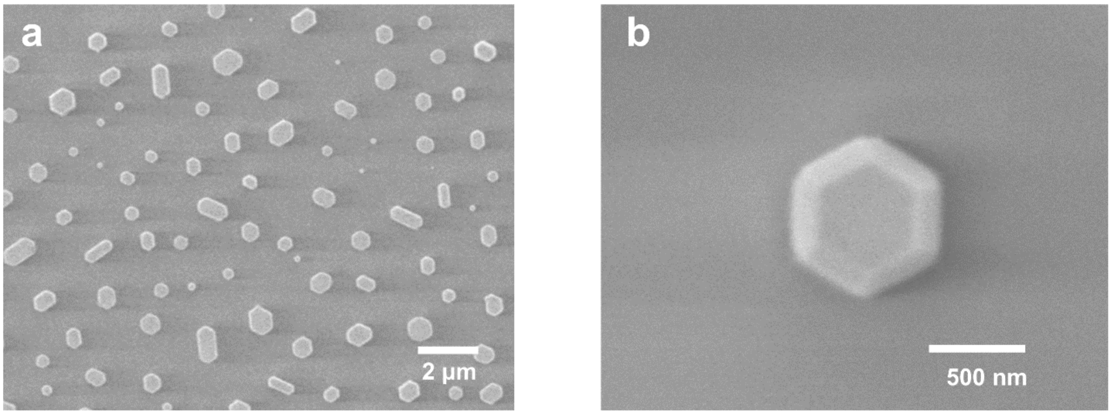

Supplementary Figure 1. Dewetted Pt nanocrystals on a sapphire substrate. The SEM images of Pt nanocrystals dewetted for 14 h at 1700 ºC. **a**, magnified 6 x 10^3 times. **b**, magnified 5 x 10^4 times.

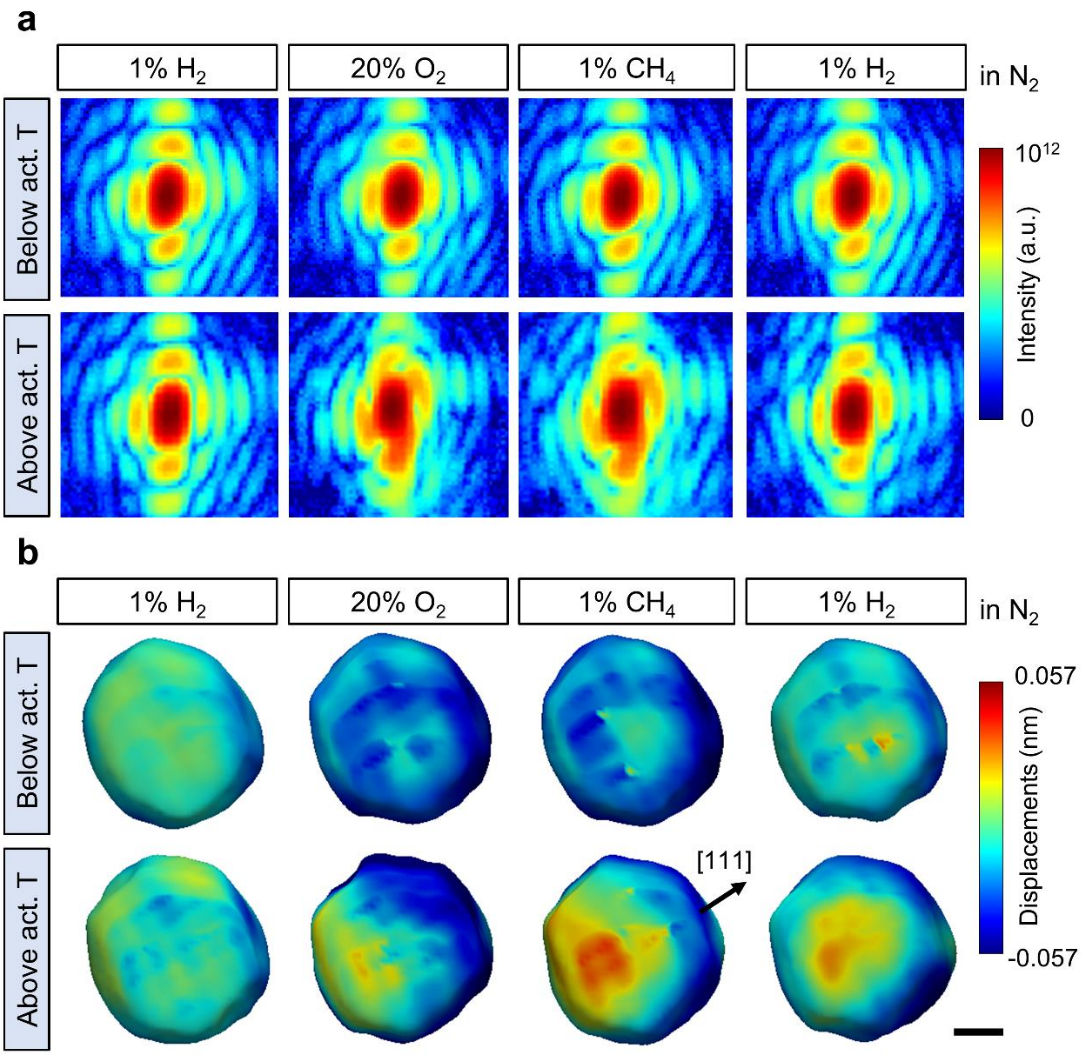

Supplementary Figure 2. Coherent x-ray diffraction patterns and 3D reconstruction images measured in various gas conditions below and above activation temperature. a, (111) Bragg CXD patterns of a 200 nm Pt particle were measured below and above activation temperature, respectively, in different gas environments. Above activation temperature, the pattern becomes distorted in 20% O_2 and undergoes even more distortion in 1% CH_4. Subsequently, it returns to the original pattern in 1% H_2. **b**, The 3D reconstruction images with 25% of isosurface acquired from CXD patterns in **a.** Red (positive sign) indicates the projected displacements along the [111] direction, u_{111}, and blue (negative sign) implies the opposite direction. The displacements at the surface with different gas environment as a function of temperature show similar trend as CXD patterns. The scale bar represents 50 nm.

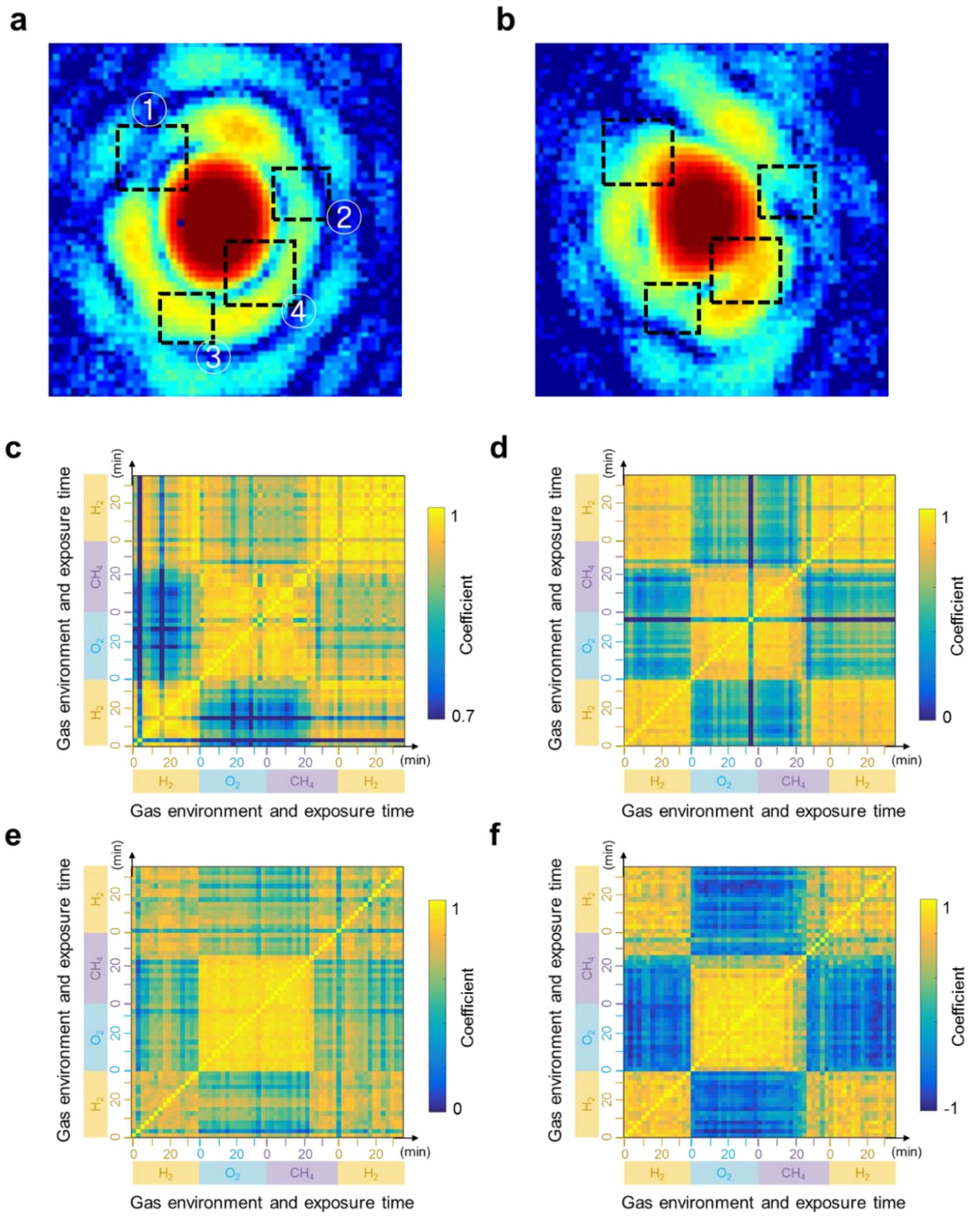

Supplementary Figure 3. CXD patterns for pristine state and oxidized state, and the cross-correlation maps for four selected regions. a-b, Four areas in dashed boxes in the first fringe region (①, ②, ③, and ④) show most differences between **a** and **b**, which are corresponded to Fig. 1a and 1b, respectively. In fact, each selected region represents the corresponding volume in the integrated 3D CXD patterns. **c-f**, The cross-correlation maps are for 3D CXD patterns within four areas, i.e., ①, ②, ③, and ④ in **a**, respectively. On the *x*- and *y*-axis, gas environments and the exposure times

are displayed. Time 0 indicates the start of each gas flow. The correlation coefficient, 1 means total positive linear correlation, 0 no linear correlation, and -1 total negative linear correlation. Since all four show similar cross-correlation patterns, their average is shown in Fig. 2c.

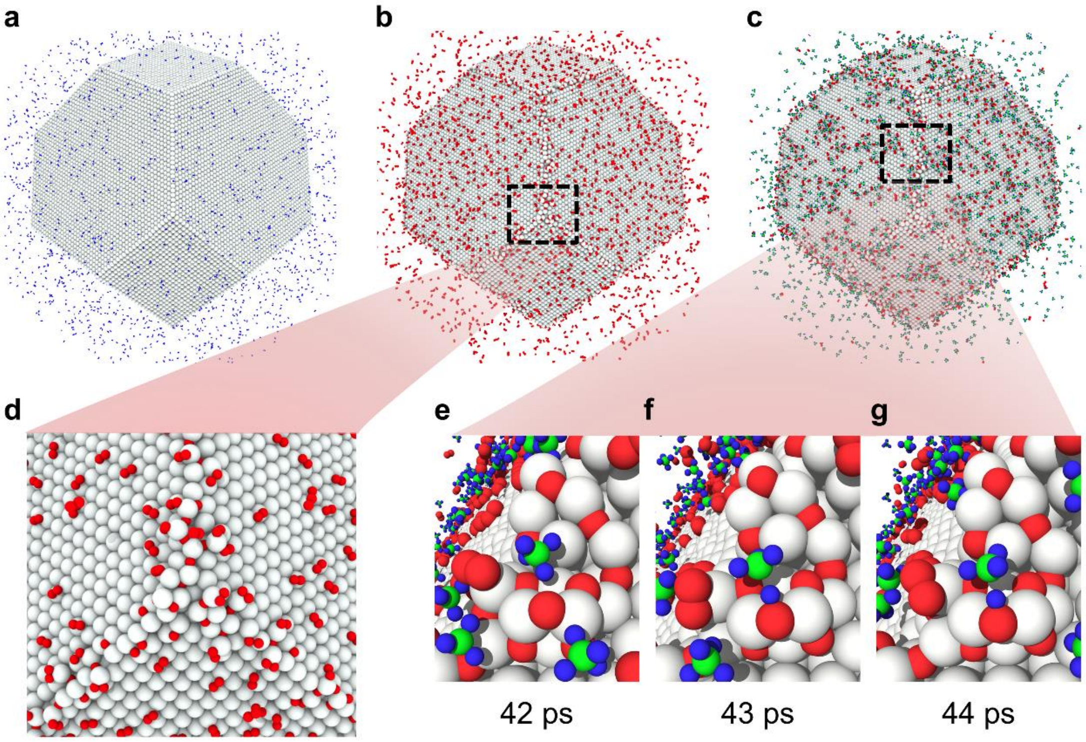

Supplementary Figure 4. Reactive molecular dynamics (RMD) simulation of Pt catalytic activity. RMD simulation was performed as described in Methods. **a-c**, A single Pt crystal with reactants of H_2, O_2, and CH_4 molecules coloured in each atom as follows: Pt atoms are in grey, O atoms in red, carbon in green and H in blue. **a**, Pt in an environment of pure H_2. **b**, Pt in an environment of pure O_2. Oxidation of Pt is seen on the edge and corner sites, while O_2 adsorbs on the faces of the particle without dissociation. **c**, Oxidized Pt nanocrystal in the presence of CH_4. **d**, close-up view of highlighted region in **b** showing O atoms as a result of O_2 molecule dissociation at the edges and the corner. **e-g**, show expanded view of region marked out in **c** showing the binding of CH_4 to an O atom on the Pt nanocrystal and oxidation of CH_4 from Δt = 42 ps to Δt = 44 ps at the edge.

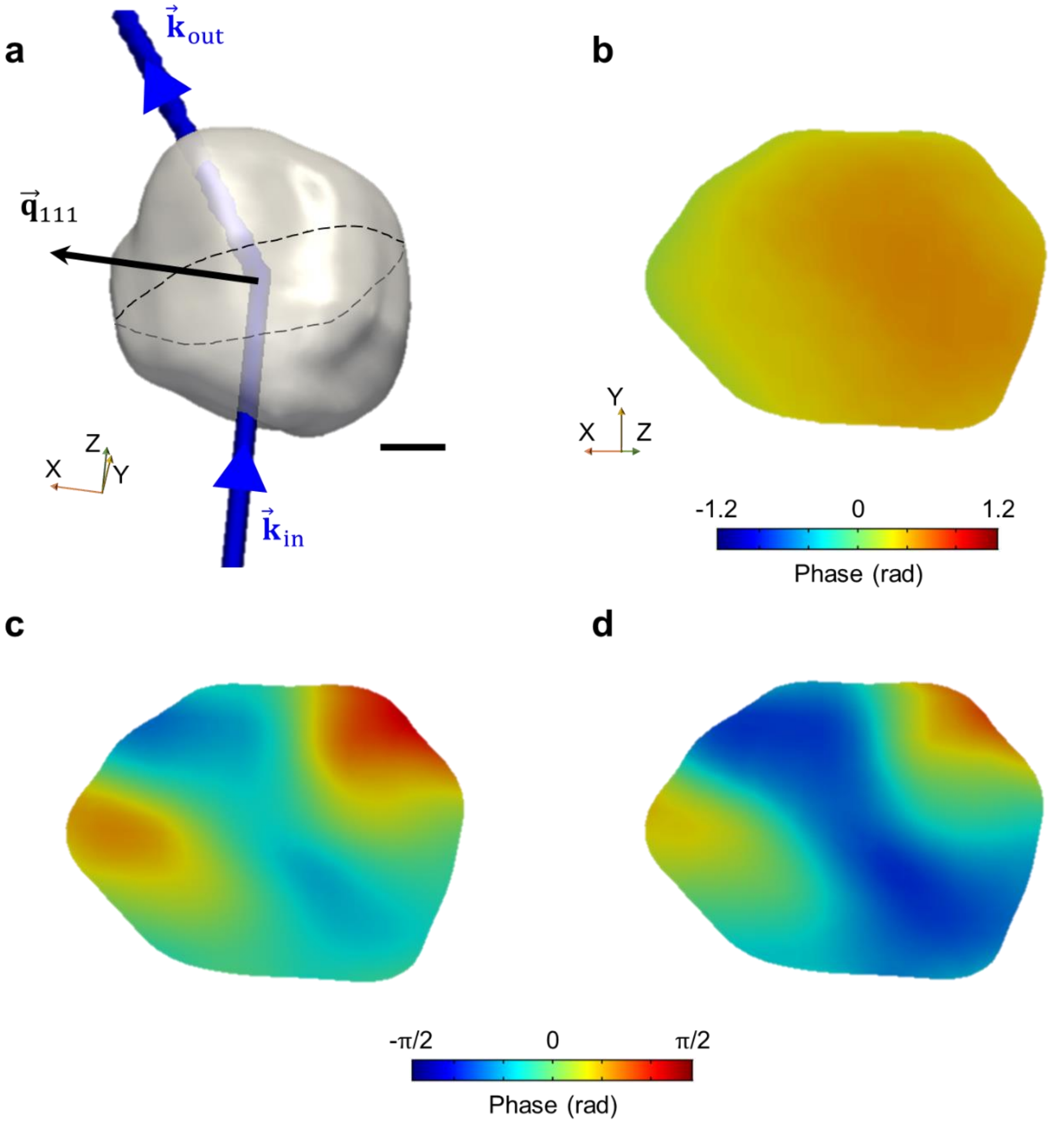

Supplementary Figure 5. Refraction correction for reconstruction 3D images. a, 25% isosurface 3D image of the Pt nanocrystal with blue arrows indicating the directions of incident and scattered x-rays, $\mathbf{k}_{in}$ and $\mathbf{k}_{out}$, respectively. The black arrow shows the total wave vector transfer ($\mathbf{q}_{111}$). **b**, The sliced image along the dashed line in **a** shows the magnitude of shifted phase calculated by $kd\delta$, where k is the wavenumber, d optical path, and δ dispersion of the refractive index[1]. **c**, The cross-section as same plane as in **b** depicts reconstructed phase map under O_2 gas flow before the correction. **d**, The cross-section after the correction. The scale bar represents 50 nm.

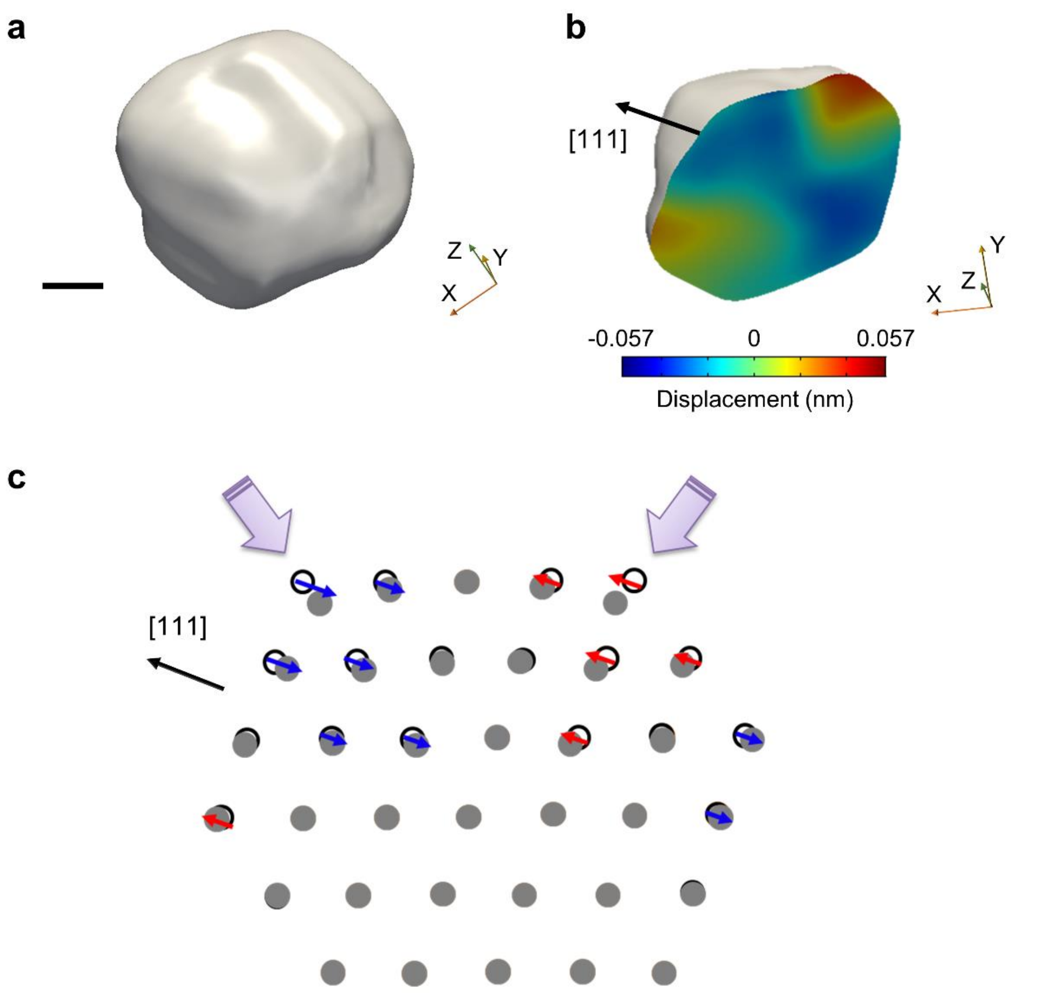

Supplementary Figure 6. The reconstructed image for the Pt nanocrystal and the simplified scheme for deformed Pt lattice. a, A 3D amplitude image of the Pt nanocrystal with 25% isosurface in O_2. **b**, The slice shows internal deformation field distribution for the same crystal. The blue (negative sign) denotes the projected displacement to the opposite direction of [111] and the red (positive sign) implies that along [111]. **c**, The simplified scheme for deformed Pt shows the lattice positions of Pt atoms representing the displacement map in **b**. Open black circles indicate original position of Pt atoms and filled gray circles depict the shifted position in the forced state where the purple arrows show the direction of forces. Difference between the original and shifted positions is indicated by red and blue arrows for displacements along the same and opposite direction of [111], respectively. Therefore, Pt atoms in O_2 show the overall contraction by the forces generated at the upper edges owing to oxygen adsorption. The scale bar corresponds to 50 nm.

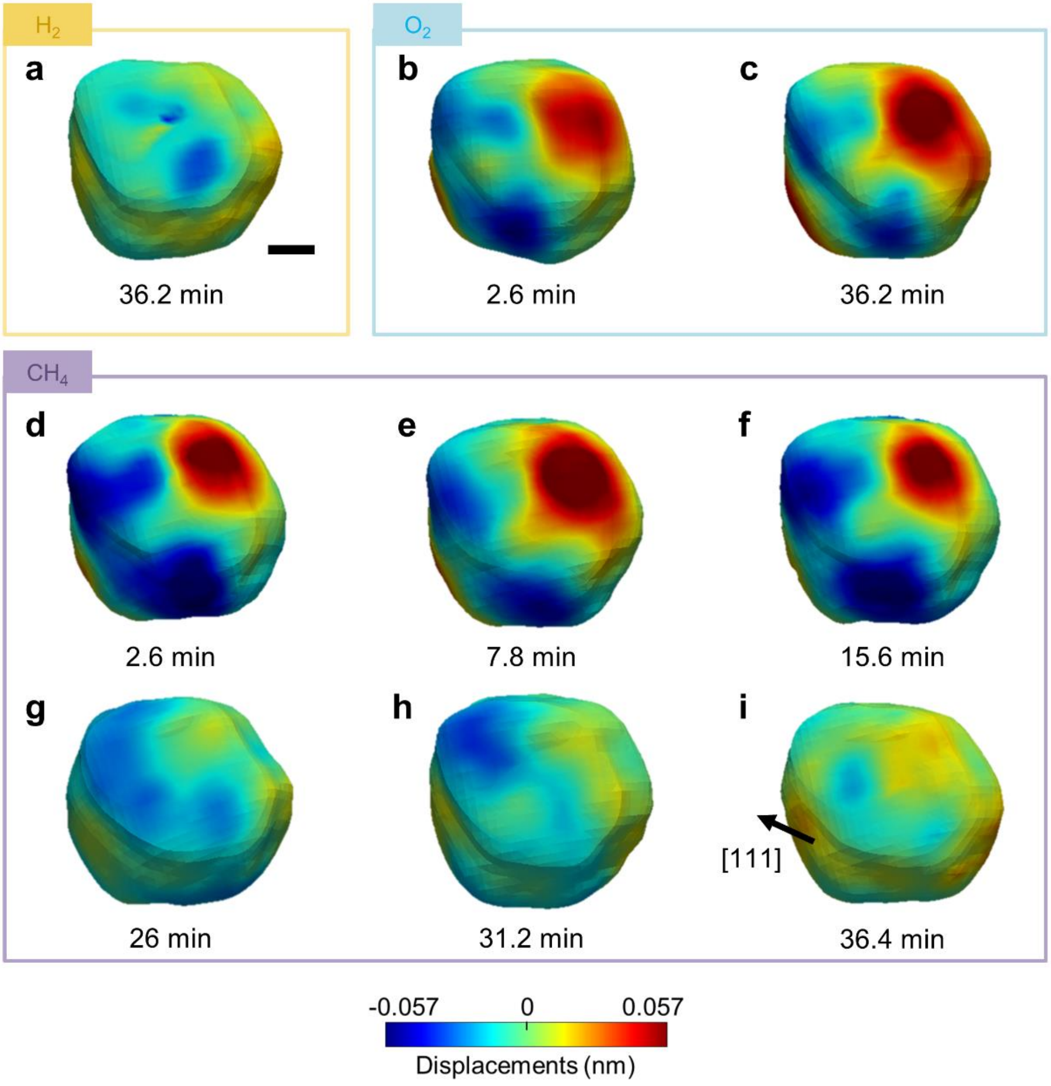

Supplementary Figure 7. Evolution of the displacements in 3D reconstructed images of the Pt nanocrystal in various gas conditions above the activation temperature. The exposure times of each gas flow are indicated below the 3D images taken under **a**, 1% H_2 gas flow, **b**-**c**, 20% O_2 gas flow, and **d**-**i**, 1% CH_4 gas flow. The initial state of the Pt nanocrystal is deformed under 20% O_2 gas flow. The deformation persists in O_2 even after ~36 min (indicating adsorption of O atoms) and continues 22 min after insertion of 1% CH_4 gas flow. Finally, it returns to the initial state after ~36 min. It is consistent with the cross-correlation map (in Fig. 2c). The scale bar represents 50 nm.

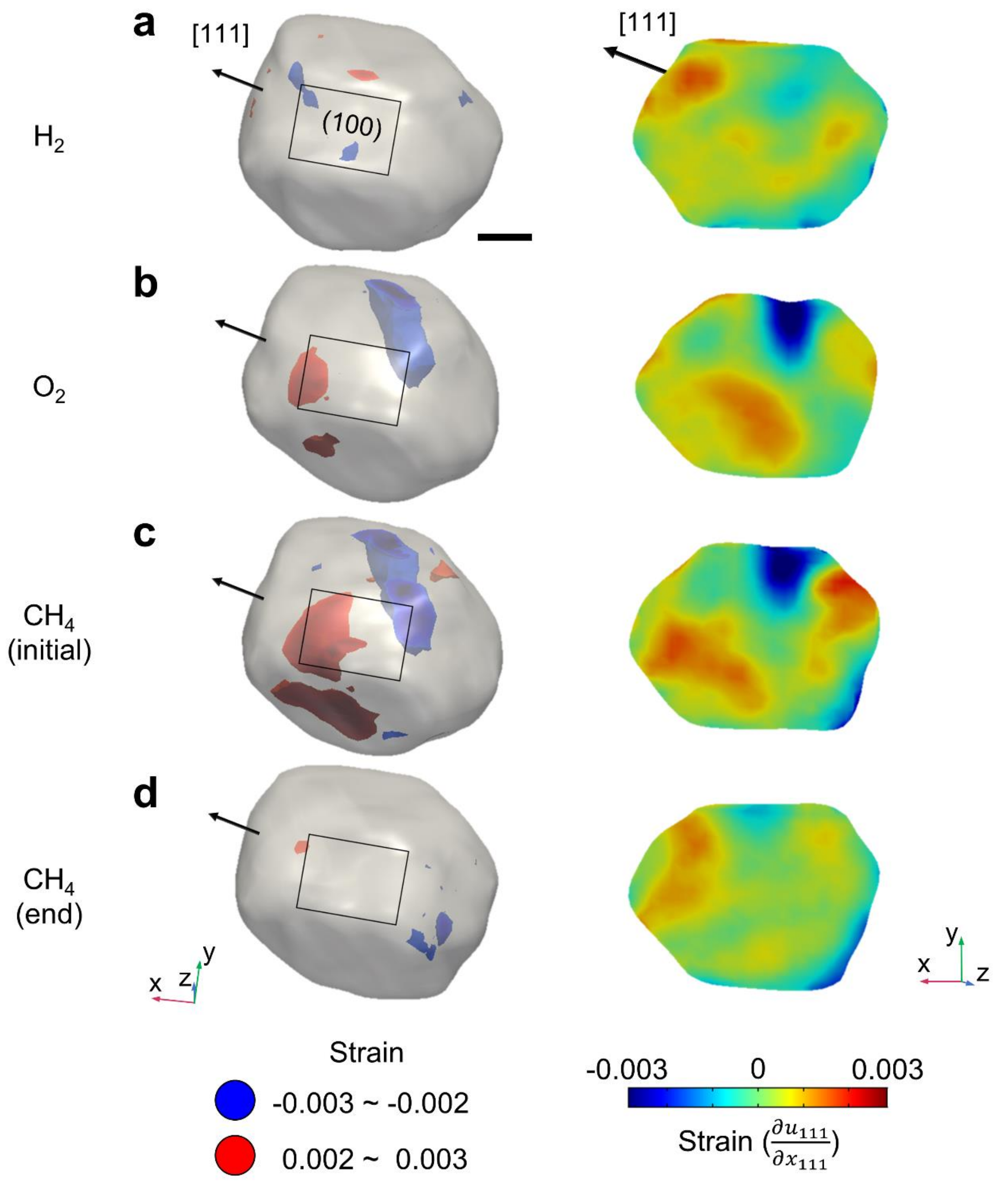

Supplementary Figure 8. Strain maps for the Pt nanocrystal with various gas environments above the activation temperature. The 3D strain maps with front view of (100) facet (on the left) and vertical slices (on the right) are shown for the reconstruction images in Fig. 4a-d. The strain evolution, calculated by derivatives of displacements along the measurement direction, is described with highly strained area ($|\partial u_{111}/\partial x_{111}| > 2 \times 10^{-3}$). **a**, The crystal in H_2 shows residual strain originated from a growth process. **b**, Under O_2 gas flow, highly strained area locates at the corner of (100) facet with tensile strain

(in red, positive values) on the left and compressive strain (in blue, negative values) on the right. The sliced image also shows compressive strain from the upper part of the crystal. **c**, The stronger strain appears under initial CH_4 flow. The image shows dominant strain along $\mathbf{q}_{111}$ direction at the edges of (100) facet, but strain components arisen from the edges of other facets can be measured at other Bragg peaks. **d**, The strain disappears after completion of methane catalytic oxidation, the strain map returns to the original state in the slice image. The scale bar represents 50 nm.

SUPPLEMENTARY REFERENCES

1. Harder, R. et al. Orientation variation of surface strain. *Phys. Rev. B* **76**, 115425 (2007).
